# Modification of large area Cu_2_O/CuO photocathode with CuS non-noble catalyst for improved photocurrent and stability

**DOI:** 10.1038/s41598-020-75700-7

**Published:** 2020-10-30

**Authors:** G. Panzeri, M. Cristina, M. S. Jagadeesh, G. Bussetti, L. Magagnin

**Affiliations:** 1 Dipartimento di Chimica, Materiali e Ingegneria Chimica Giulio Natta, Politecnico di Milano, 20131 Milan, Italy; 2 Dipartimento di Fisica, Politecnico di Milano, 20133 Milan, Italy

**Keywords:** Chemistry, Energy science and technology, Nanoscale materials, Engineering, Chemical engineering, Materials science, Materials for devices, Materials for energy and catalysis

## Abstract

In this work, a three-layered heterostructure Cu_2_O/CuO/CuS was obtained through a low-cost and large-area fabrication route comprising electrodeposition, thermal oxidation, and reactive annealing in a sulfur atmosphere. Morphological, microstructural, and compositional analysis (AFM, SEM, XRD, EDS, XPS) were carried out to highlight the surface modification of cuprous oxide film after oxidation and subsequent sulfurization. Impedance, voltammetric, and amperometric photoelectrochemical tests were performed on Cu_2_O, Cu_2_O/CuO, and Cu_2_O/CuO/CuS photocathodes in a sodium sulfate solution (pH 5), under 100 mW cm^−2^ AM 1.5 G illumination. A progressive improvement in terms of photocurrent and stability was observed after oxidation and sulfurization treatments, reaching a maximum of − 1.38 mA cm^−2^ at 0 V versus RHE for the CuS-modified Cu_2_O/CuO electrode, corresponding to a ~ 30% improvement. The feasibility of the proposed method was demonstrated through the fabrication of a large area photoelectrode of 10 cm^2^, showing no significant differences in characteristics if compared to a small area photoelectrode of 1 cm^2^.

## Introduction

Cuprous oxide (Cu_2_O) is one of the most investigated p-type semiconductor material in the framework of photoelectrochemical hydrogen production^1–3^, mainly because of its optical properties and potentially low-cost synthesis^4^. It has indeed a direct bandgap of ~ 2.1 eV, capable to absorb a large part of the solar spectrum, resulting in a theoretical maximum photocurrent density of − 14.7 mA cm^−2^ at AM 1.5 G condition^4^, and has a favorable conduction band position with respect to the hydrogen evolution reaction potential. Moreover, the simple chemical composition, based on earth-abundant elements, allows its synthesis through many different routes (hydrothermal synthesis^5,6^, thermal oxidation^7^, sputtering^8,9^, electrodeposition^10^), and to achieve a large variety of architectures (micro and nano-crystals^5,6,11^, thin-films^8–10^, nanowires^12,13^). For thin-film based devices, electrodeposition is the major candidate towards the development of low-cost and large-area production and it has been already exploited to achieved efficiency records during the last decade for Cu_2_O-based photoelectrodes^4,14,15^. However, the main drawback affecting this material is the poor stability against photodegradation. In fact, upon illumination, electrons reach the solid–liquid interface reducing the semiconductor to the metallic state (Cu_2_O → Cu) progressively decreasing the photocurrent generated^16^. To minimize this phenomenon, the formation of heterostructures, together with the implementation of a catalyst, is a common and one of the most effective strategy. However, the most performing overlayers often rely on poorly scalable or expensive fabrication techniques such as atomic layer deposition (ALD)^4,14,15^, allowing the deposition of a few-nanometer thick layer, highly conformal and optically transparent. A different approach relies on the exploitation of simple and affordable routes such as thermal or chemical treatments that would result in the formation of the different heterostructures (Cu_2_O/C^12^, Cu_2_O/CuO^17–25^, Cu_2_O/CuS^26^, Cu_2_O/TiO_2_^27^, Cu_2_O/NiO^28^). Among those, one of the simplest yet effective is the oxidation of Cu or Cu_2_O, through which a surface layer of cupric oxide CuO (~ 1.3–1.8 eV bandgap) is obtained, forming a staggered band alignment with the underneath cuprous oxide. Besides improving the charge transfer, i.e. reducing recombination, Cu_2_O/CuO heterostructure showed higher stability than the bare Cu_2_O^18,20^, further improved with the application of catalysts such as Ni, Pt, and CuS^18,21^. In particular, Cu_2_O/CuO/CuS photoelectrode showed remarkable characteristics such as a photocurrent density at 0 V versus RHE of − 5.4 mA cm^−2^ with an 82% stability over 1-h test^21^. However, in that study, the researchers relied on a time consuming and impractical deposition technique for the catalyst CuS layer such as the selective ionic layer adsorption reaction (SILAR) method^21^. In the present study, we demonstrate the possibility to obtain Cu_2_O/CuO/CuS heterostructure through a three steps process involving only low cost and potentially large scalable techniques such as electrodeposition and thermal treatments.

## Experimental

Cu_2_O was electrodeposited on FTO substrate at − 0.1 mA cm^−2^ in a thermostatic bath at 40 °C. The solution consisted of 0.3 M of copper sulfate (Sigma-Aldrich, purity 99.8%) and 3 M of lactic acid (Sigma-Aldrich, 85% w/w), brought to pH 11 with NaOH (Sigma-Aldrich, purity 99.9%). Thermal oxidation was subsequently carried out in an open tubular furnace at 400 °C for 0.5–2 h. Reactive annealing was performed in a sulfur atmosphere (10 mg S), using N_2_ carrier gas, at 300 °C for 300 s. The quartz vial was firstly cleaned with flowing nitrogen (3 Nl h^−1^) and subsequently inserted in the preheated furnace. Scanning electron microscopy (SEM) (Zeiss EVO 50 EP), tapping-mode atomic force microscopy (AFM), and electron dispersive spectroscopy (EDS) (Oxford instruments INCA x-sight detector) were carried out to assess the morphology and composition of the heterostructure, along with X-ray diffraction analysis (XRD) (Bragg–Brentano), used for the microstructural investigation (Philips model PW1830. K_a1_Cu = 1.54058 Å). Glow discharge optical emission spectroscopy (GDOES) was performed at 700 V using Ar gas (0.23–0.36 kPa) (Spectrum Analytic GmbH GDA 750 analyzer). The X-ray photoelectron spectroscopy (XPS) measurement was performed, in an ultra-high vacuum (UHV) chamber with the base pressure of 2 × 10^–10^ Torr, with an un-monochromatized Mg Kα source (photon energy = 1253.6 eV) at normal emission and room temperature. The photoelectrons were collected by a 150 mm hemispherical analyzer (SPECS GmbH) with a pass energy of 20 eV for high-resolution spectra and 40 eV for wide spectra. The overall FWHM resolution of the combined photon source and the spectrometer was below 1 eV. A polycrystalline Au sample was used to calibrate the Fermi level. Photoelectrochemical characterization and testing were performed in a three-electrode cell comprising Ag/AgCl as a reference electrode and a Ru-based mixed metal oxide (MMO) net as a counter for both linear sweep voltammetry (LSV) and amperometric test (Amel 2559 potentiostat/galvanostat). Electrochemical impedance spectroscopy (EIS) was performed at + 0.3 V versus RHE, under illumination (1 sun) 1–10 MHz with 5 mV AC amplitude (Admiral Instruments, Squidstat PLUS). A 0.5 M Na_2_SO_4_ solution at pH 5 was used for the photoelectrochemical tests. The light source used was a 100 mW cm^−2^ AM 1.5 G solar simulator (ABET 11002 SunLite).

## Results and discussion

### Cu_2_O/CuO heterostructure

**Figure 1:**
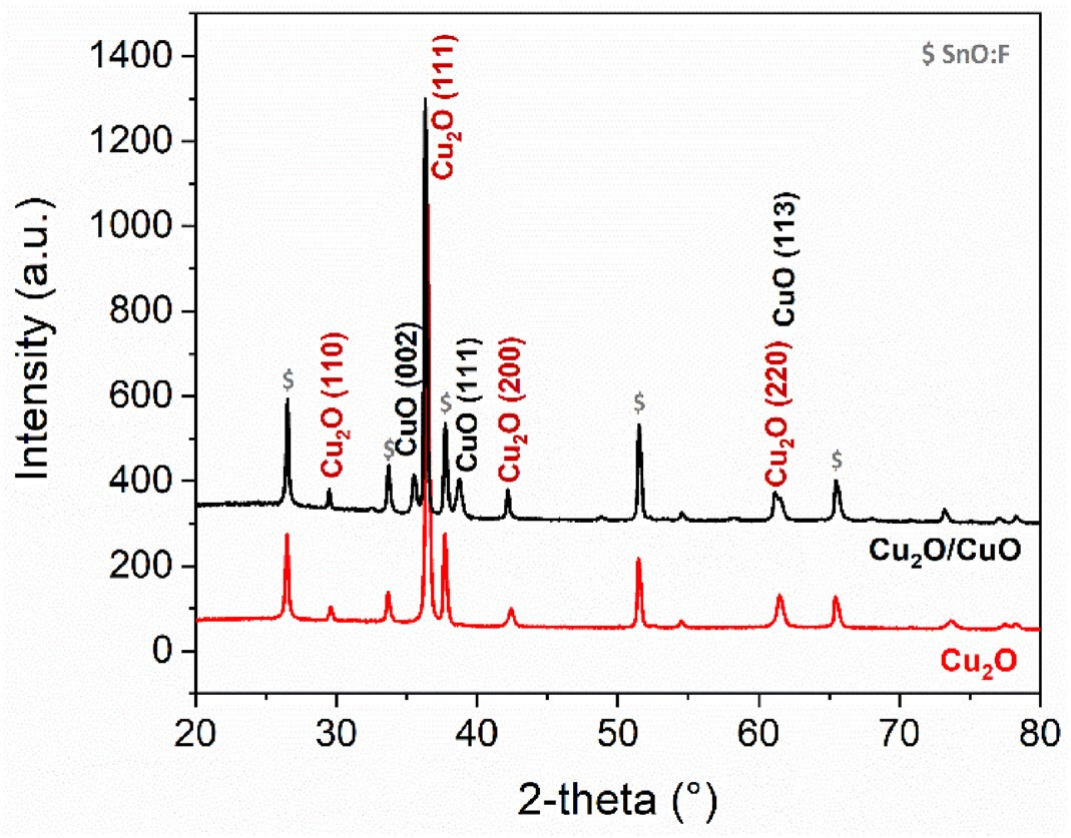
XRD patterns of FTO/Cu_2_O and FTO/Cu_2_O/CuO photoelectrodes.

Cu_2_O films were prepared by electrodeposition using a well-known lactate stabilized solution^10^. While the majority of the studies report the growth of cuprous oxide on Au-sputtered substrate at relatively high pH values (pH ~ 12–12.5)^29^, on FTO the plating conditions vary more among different literature works^30^. Preliminary tests were carried out at varying pH (9–12), keeping constant the bath formulation, the deposition temperature (40 °C), and the current density (− 0.1 mA cm^−2^). The desired microstructure was found growing the film onto the FTO substrate, at pH 11 (Fig. 1). The intensity of the different peaks revealed the film to be preferentially oriented along the (111) direction, whose diffraction peak was predominant. Theoretically, (111) is the most interesting orientation from the charge transfer viewpoint since Cu_2_O conductivity is attributed to the presence of point defects such as O vacancies^30^, resulting in a higher photoelectrochemical activity^31^.

**Figure 2:**
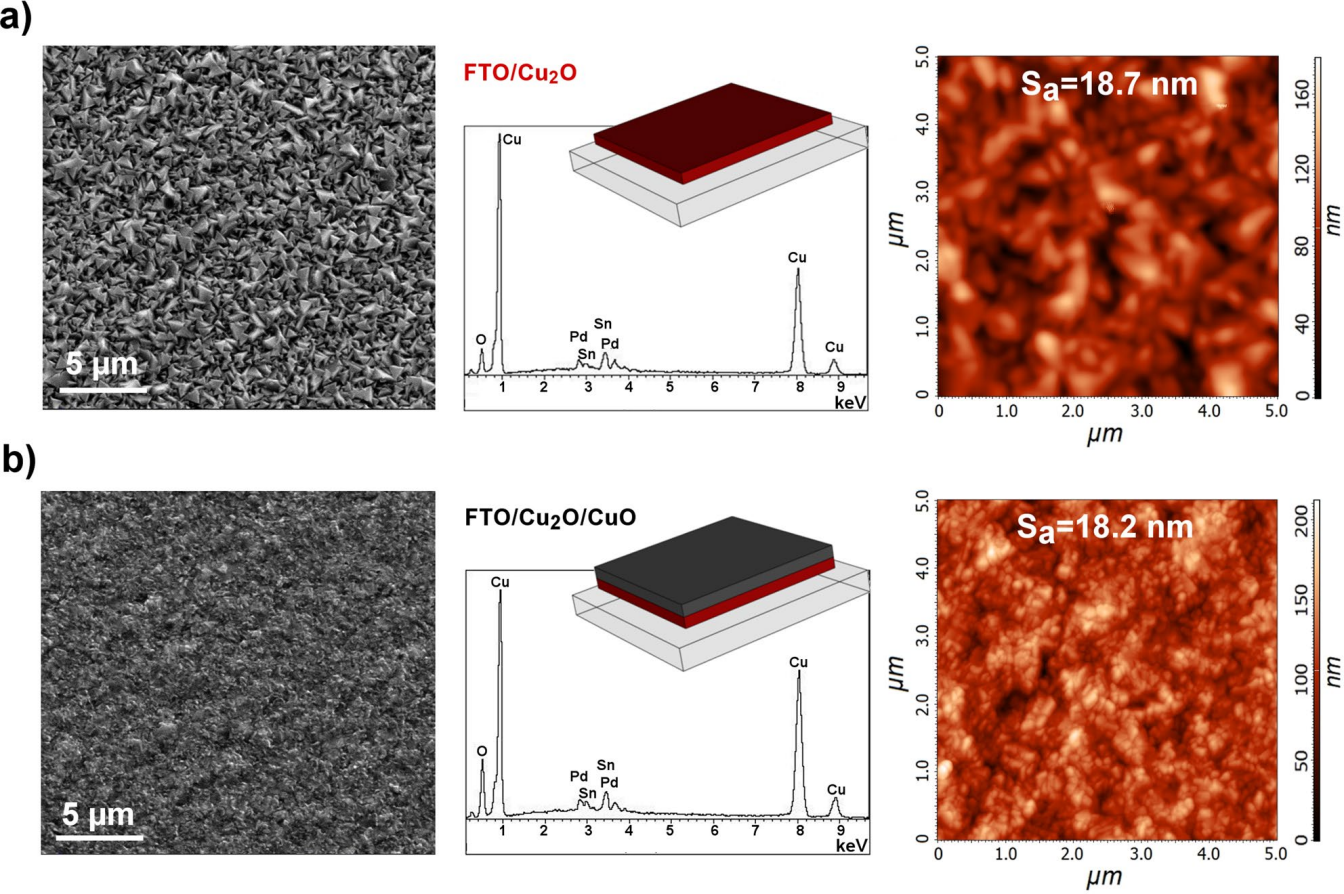
SEM (scale bar: 5 µm), EDS and AFM of (**a**) FTO/Cu_2_O and (**b**) FTO/Cu_2_O/CuO photoelectrodes.

In agreement with XRD patterns (Fig. 1), SEM micrographs of Cu_2_O films showed a truncated pyramid shape morphology typical of (111) plane of the cubic cell (Fig. S1, Fig. 2a). The oxide characteristic morphology became clearer and more distinct as the deposition charge density increases (≥ 0.75 C cm^−2^) (Fig. S1) while lower deposition time resulted in a more compact surface, with a finer structure and lower surface roughness. A progressive increase in the dimension of the crystallites size was observed for longer deposition times, as a result of the growth of the Cu_2_O nuclei going from the nanometer to the micrometer scale (Fig. S1). Theoretically, large-grained material is highly desirable from a semiconducting viewpoint, a more ordered and less-defected structure would result in more efficient charge separation and extraction since recombination is more likely to occur at defects.

After air annealing, the XRD pattern showed well-defined peaks associated with cuprous and cupric oxide^32^, in agreement with the formation of the heterojunction (Fig. 1). The preferential orientation of the Cu_2_O underlayer was maintained, as shown by the major diffraction peak Cu_2_O (111), while the CuO phase appeared to be randomly oriented as indicated by the relative intensity of (002), (111), (113) diffraction peaks (Fig. 1, Fig. S2). As expected, by increasing the annealing time a larger portion of the material was oxidized to cupric oxide (Fig. S3), maintaining the layered structure. EDS results were in agreement with XRD data, detecting the formation of a phase richer in oxygen, as evinced by the relative peak heights respectively for Cu and O (Fig. 2a,b). Surface micrographs of Cu_2_O/CuO samples showed a significant change in the morphology, characterized by a fine structure and a lower surface roughness, as confirmed by AFM analysis (Fig. 2a,b). It is worth to remark that the quality of the Cu_2_O/CuO heterojunction was dependent on the underneath Cu_2_O, not only in terms of the structure at the microscale (roughness) but especially at the macroscopic one. The sample homogeneity at the macroscale was found to be greatly affected by the electrodeposition charge density, the formation of macroscopic defects (holes) in the layer was indeed directly correlated to the thickness of the cuprous oxide, the thicker the layer the lower the surface homogeneity after oxidation. On the other hand, for charge densities ≤ 1 C cm^−2^ no macroscopic defects were detected. This was hypothesized to be related to the stresses involved during the oxidation process and the formation of the heterostructures, small delaminated powdery material, coming from the sample, could be found in the quartz vials in case of a thick cuprous oxide film.

### Cu_2_O/CuO/CuS heterostructure

**Figure 3:**
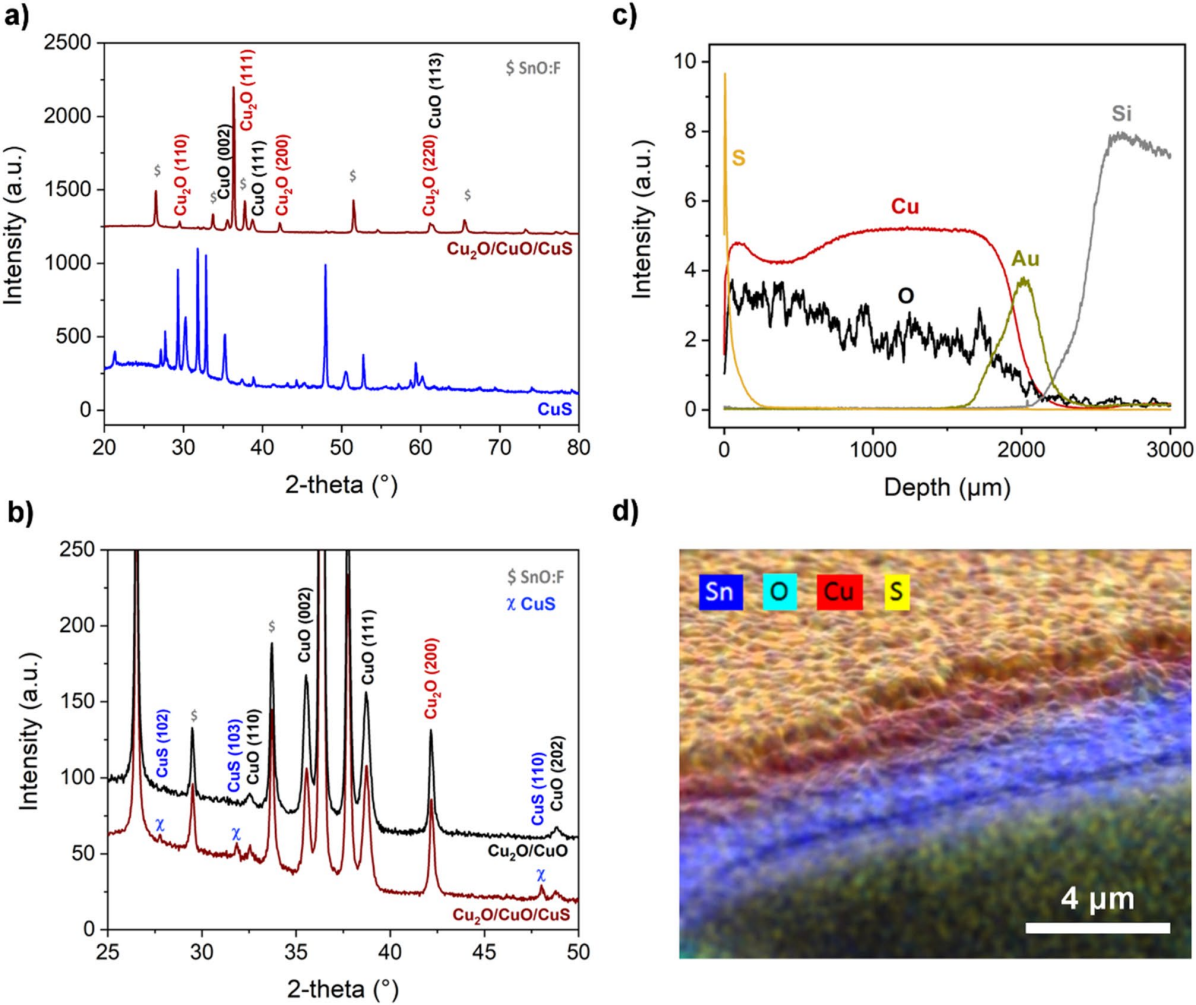
XRD patterns of (**a**) FTO/Cu_2_O/CuO/CuS photoelectrode and overreacted FTO/CuS film. (**b**) comparison between FTO/Cu_2_O/CuO/CuS and FTO/Cu_2_O/CuO photoelectrodes. (**c**) GDOES elemental profile of Si(wafer)/Au/Cu_2_O/CuO/CuS heterostructure. (**d**) SEM micrograph with EDS elemental mapping of fractured FTO/Cu_2_O/CuO/CuS photoelectrode.

**Figure 4:**
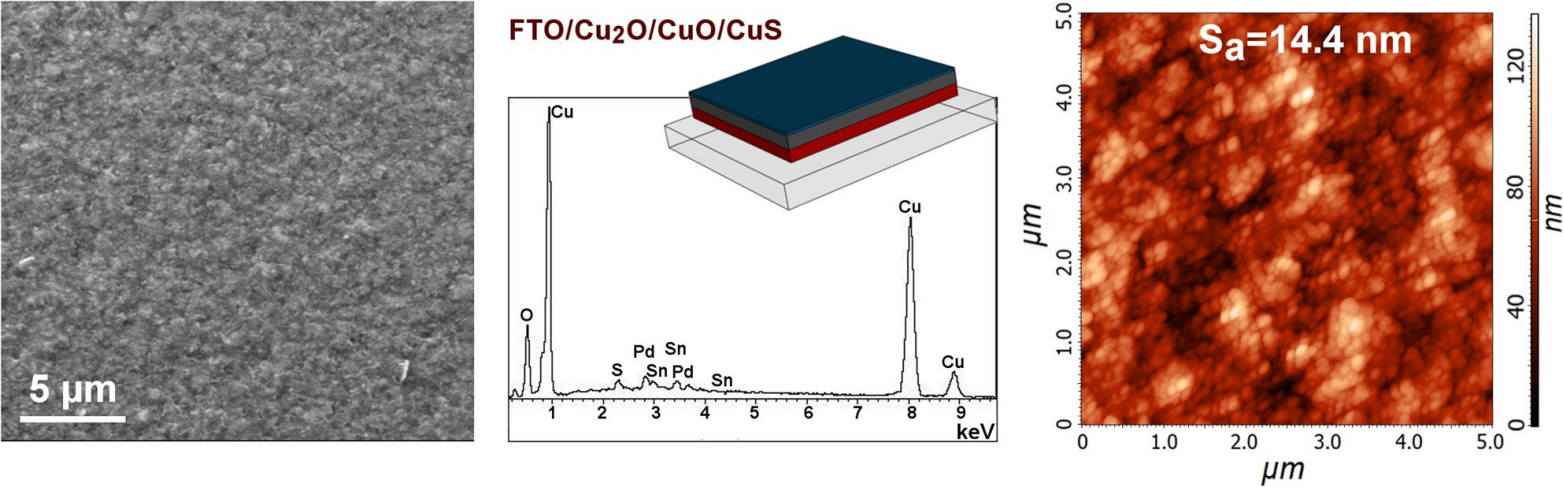
SEM (scale bar: 5 µm), EDS, and AFM of FTO/Cu_2_O/CuO/CuS photoelectrode.

Copper sulfide (CuS) overlayer was grown on Cu_2_O/CuO by reactive annealing in a sulfur-containing atmosphere, using nitrogen as a gas carrier. The sulfurization of the oxidized samples was investigated in a relatively narrow temperature interval (300–400 °C) after preliminary investigation. Specifically, an appreciable reactivity was found for temperatures higher than 250 °C while an upper limit has been set due to the low boiling point of sulfur (~ 444 °C). Above such a temperature, sulfur would evaporate quickly providing an irregular and unpredictable supply of gaseous sulfur species on the sample surface during the test. At 400 °C, the reactivity was found to be too high since a short amount of time (~ 3 min) min was enough to convert both the oxide layers into CuS, nonetheless, the optimal reaction time was found to be 2 min. However, to better control the growth of the top sulfide layer, the temperature was reduced to 300 °C and the reaction time extent to 5 min. It is important to notice that the reactivity of Cu_2_O is much greater than CuO; if exposed, the underneath Cu_2_O would quickly react having no control over the process. The homogeneity and coverage of the upper CuO layer are thus crucial for the formation of a three-layered heterostructure. Because of that, particular attention was paid to the optimization of the furnace setup, allowing to carry out surface treatments homogeneously. For the sulfurization step, the samples were placed at the far end of a one-hand closed quartz tube (3 cm diameter) placing elemental sulfur upstream at low nitrogen flux (3 Nl h^−1^). The formation of Cu_2_O/CuO/CuS came along with a slight color change into a golden-brownish appearance, while for an overreacted sample the color would result in a deep blue, typical of bulk CuS. XRD analysis detected only small peaks belonging to the covellite phase, suggesting the formation of a thin overlayer (Fig. 3a,b) along with the slight color change. The GDOES compositional profile along the z-axis confirmed the formation of the three-layered heterostructure showing three well-separated layers (Fig. 3c). The sulfur signal was only detected corresponding to the surface, indicating that the sulfurization involved only the cupric oxide layer. On the other hand, the copper and oxygen signals varied along with the thickness in agreement with the Cu_2_O/CuO structure where the oxygen-poor phase was found in the proximity of the substrate (Fig. 3c). EDS elemental mapping (Fig. 3d, Fig. S4) of the fractured surface of the sample further confirmed the compositional stratification, in agreement with XRD and GDOES results. The sulfurized surface was similar to the oxidized sample, showing to be compact and with a small-grained morphology characterized by oval clusters, a further reduction in the average surface roughness was observed (Fig. 4, Fig. S5).

**Figure 5:**
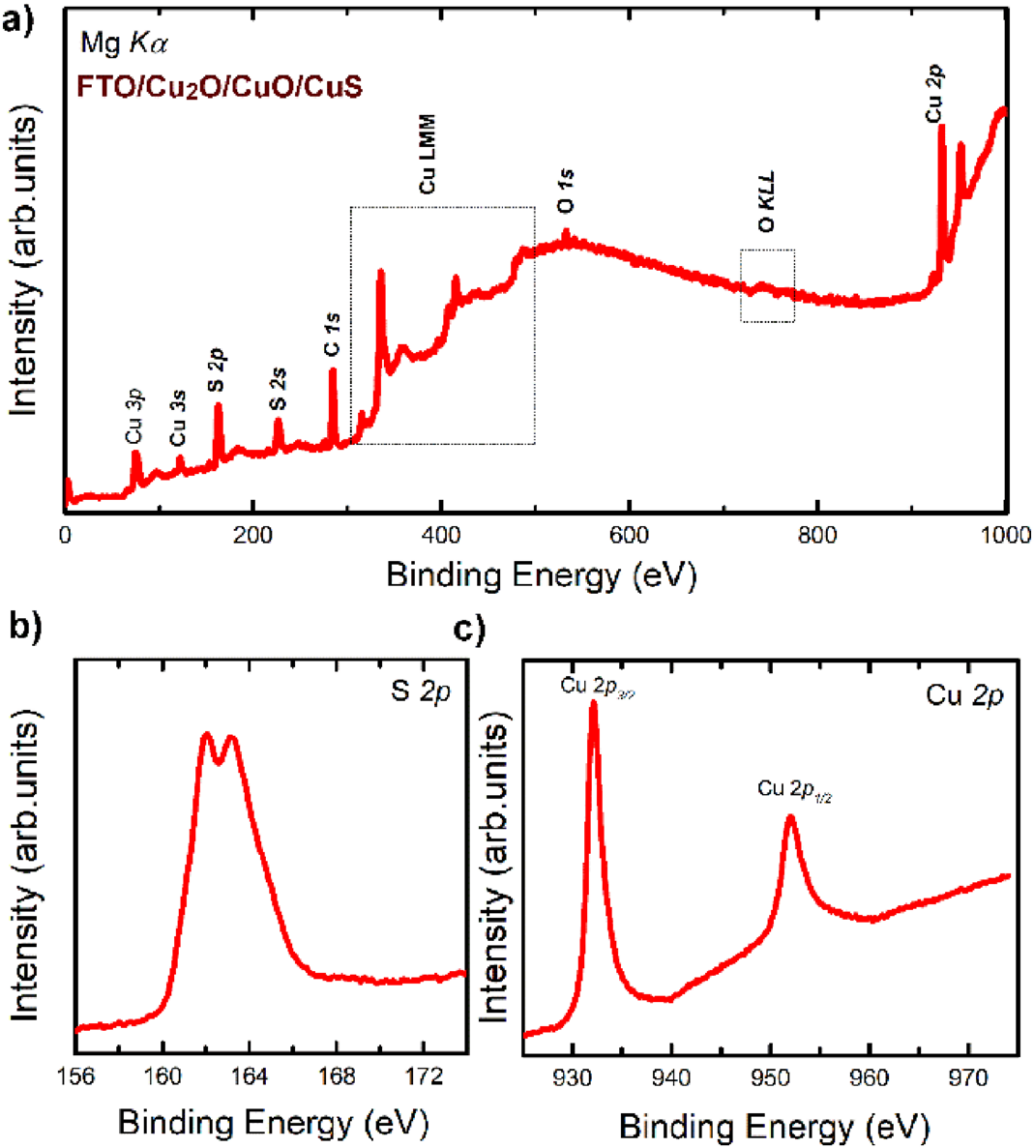
(**a**) XPS survey of Cu_2_O/CuO/CuS. (**b**) XPS S *2*p of Cu_2_O/CuO/CuS. (c) the Cu *2*p region of the XPS spectra of Cu_2_O/CuO/CuS.

To assess with more accuracy the surface elemental composition, samples were analyzed by XPS. The survey spectra of the sample Cu_2_O/CuO/CuS has been reported in Fig. 5a, where copper, sulfur, and oxygen characterized the sample composition. Being introduced from outside the measurement chamber and considering adventitious hydrocarbon from XPS, also a small carbon feature was detected. The intensity of both Cu and S peaks were significantly higher compared to the O one, confirming the presence of CuS in the uppermost layers of the sample, while Cu_2_O and CuO were buried. The main peak of the Cu Auger LMM lines (dotted box in the figure) had a BE = 336 eV, which is compatible with an oxidation state equal to + 2. From the analysis of the survey spectrum, we deduced the formation of the CuS compound. However, the chemical state of these elements was better studied from a high-resolution XPS analysis (Fig. 5b,c). The binding energy position of Cu 2*p*_3/2_ and Cu 2*p*_1/2_ (932.1 eV and 952.1 eV, respectively) S 2*p*_3/2_ and S 2*p*_1/2_ (162.1 eV and 163.2 eV, respectively) were in good agreement with the literature^21,33^. The symmetrical behavior of copper peaks and the measured sulfur binding energy confirmed the formation of CuS. Regarding the copper oxide buried layers, we noted that the very weak shoulder at around 943 eV, where the Cu shake-up feature is generally observed, also suggested that the oxide layers were below the CuS one^21^.

### Photoelectrochemical characterization

**Figure 6:**
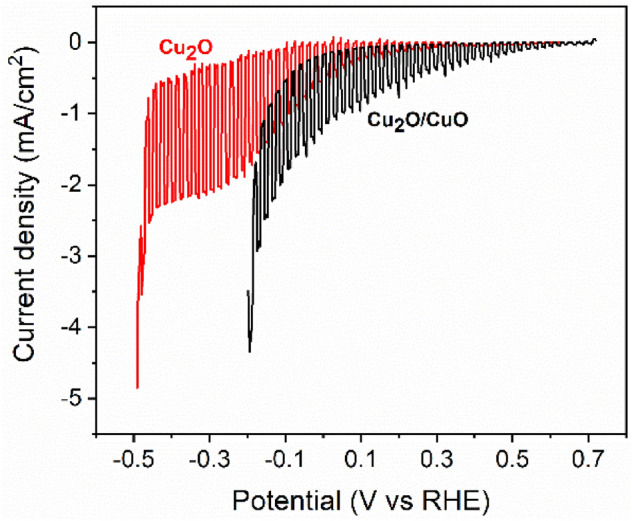
LSV of Cu_2_O and Cu_2_O/CuO photoelectrodes in 0.5 M Na_2_SO_4_ at pH 5.

Photoelectrochemical voltammetry tests were carried out to evaluate the semiconductor behavior at increasing cathodic bias. All the tests were performed in a 0.5 M Na_2_SO_4_ solution at pH 5 and the potential values recorded were converted to the reversible hydrogen electrode (RHE) V_RHE_ = V + V_Ag/AgCl (3 M)_ + (0.059·pH). Cuprous oxide photoelectrode showed the typical curve reported in the literature, characterized by a significant reductive photocurrent (p-type conductivity) at high cathodic bias but with a relatively small immersion potential under illumination and, more importantly, an onset potential close to the hydrogen evolution reaction one. The photoelectrochemical characterization was carried for Cu_2_O films having a relatively wide range of charge deposition density (0.5–1.25 C cm^−2^) (Fig. S6) where no significant variations were reported in the immersion potential under illumination, indicating the same chemical/phase composition. However, the 0.75 C cm^−2^ sample delivered the highest photocurrent, about − 2 mA cm^−2^ at − 0.3 V versus RHE. Whether for lower deposition time the thickness of the cuprous oxide film could not be enough for efficient absorption of the solar irradiation, for longer deposition time i.e. the large volume of material, charge recombination is more likely to increase. Thus, a deposition charge density of 0.75 C cm^−2^ was considered for the Cu_2_O layer, also because of its ability to form a homogeneous CuO layer during air annealing, as previously discussed. Although the cuprous oxide film showed a relatively high immersion potential under the illumination + 0.62 V versus RHE, significant photocurrent density values (− 0.1 mA cm^−2^) are only recorded for potential lower than + 0.31 V versus RHE, while achieving a value of − 0.53 mA cm^−2^ at 0 V versus RHE. On the other hand, the Cu_2_O/CuO heterostructure showed a clear increase in the immersion potential under illumination (~ 90 mV) along with a more positive onset potential (+ 0.55 V vs RHE at − 0.1 mA cm^−2^) due to the formation of a staggered band structure^24^, improving the charge separation (Fig. 6). As a consequence, the photocurrents recorded at low cathodic bias were much higher than the bare cuprous oxide, reaching − 1.06 mA cm^−2^ at 0 V versus RHE after annealing in the air for 2 h. Carrying out a shorter oxidation treatment (1 h) showed no variation in the immersion potential, indicating that the cupric oxide layer grew homogeneously all over the surface only after 1 h of air annealing; nevertheless, the photocurrent recorded was lower (− 0.75 mA cm^−2^ at 0 V vs RHE) (Fig. S7), in agreement with previous reports^18,21^. It is also worth to notice that, in the case of Cu_2_O, the majority of the current recorded was correlated to the reduction of the semiconductor material to the metallic state (Cu_2_O → Cu), sustaining the photocurrent densities at a high cathodic bias (< 0 V vs RHE). The energy level of the self-reduction reaction lies indeed between the conduction band and the hydrogen evolution energy level, favoring the degradation reaction rather than the proton reduction^3^. However, the dark current of the Cu_2_O/CuO photoelectrode was different from zero, especially approaching 0 V versus RHE, suggesting either a sign of catalytic activity of the overlayer or the presence of a secondary reaction such as self-reduction.

**Figure 7:**
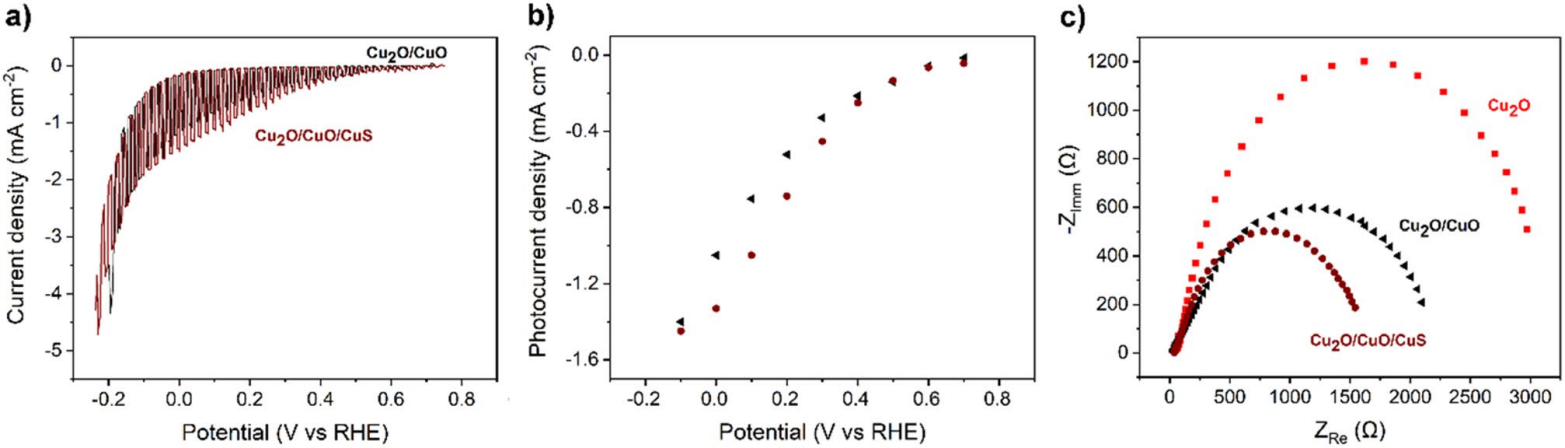
LSV of Cu_2_O/CuO and C_u2_O/CuO/CuS photoelectrodes in 0.5 M Na_2_SO_4_ at pH 5 [0.5 Hz chopped illumination at 100 mW cm^−2^ AM 1.5 G]. (**a**) Curves under chopper illumination. (**b**) Photocurrent density values defined as the difference in current density between dark and light conditions. (**c**) Nyquist plot of Cu_2_O, Cu_2_O/CuO and Cu_2_O/CuO/CuS photoelectrodes in 0.5 M Na_2_SO_4_ at pH 5 [+ 0.3 V vs RHE, 100 mW cm^−2^ AM 1.5 G].

The overall photoelectrode performances were further improved with the addition of the copper sulfide overlayer (Fig. 7a,b). The immersion potential under illumination was shifted towards more positive values at + 0.74 V versus RHE and the maximum photocurrent recorded was increased while no significant changes were observed in the curve profile. At 0 V versus RHE, the Cu_2_O/CuO/CuS heterostructures showed a photocurrent of − 1.38 mA cm^−2^, corresponding to ~ 100% and ~ 30% enhancement with respect to Cu_2_O and Cu_2_O/CuO respectively. Although no huge variations were observed in the immersion and onset potentials, a significant increase in the photocurrent was observed at moderate biases, from + 0.3 to 0 V versus RHE (Fig. 7b), when compared to the oxidized sample. Copper sulfide is thus expected to act as a catalyst, improving the charge transfer from the semiconductor surface to the electrolyte, as reported by a previous literature work^21^. To highlight the role of CuS overlayer, the charge transfer of the photogenerated electrons was studied by electrochemical impedance spectroscopy (EIS) at + 0.3 V versus RHE, under illumination (Fig. 7c). The typical semicircular feature of the Nyquist plot was found for all the three photoelectrodes, showing a progressive reduction in the semicircle diameter, associated with the charge transfer resistance (R_ct_), passing from Cu_2_O to Cu_2_O/CuO and Cu_2_O/CuO/CuS heterostructures, confirming the staggered band alignment between Cu_2_O and CuO^17,18,24^ and the catalytic activity of CuS^21,26,33^. The results are in agreement with the photoelectrochemical polarization tests where higher photocurrents were recorded for the CuS-modified surface.

**Figure 8:**
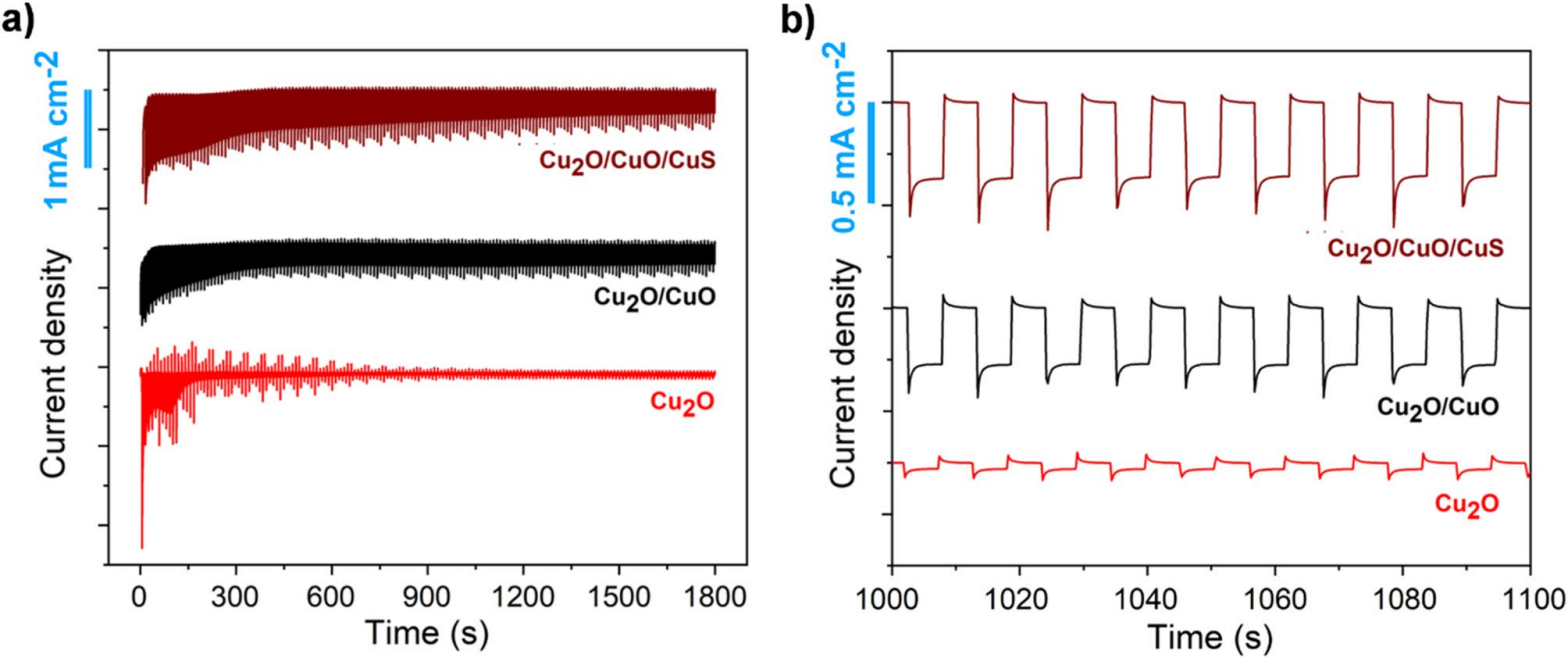
Photoelectrochemical test at a fixed potential (0 V vs RHE) under chopper illumination for FTO/Cu_2_O, FTO/Cu_2_O/CuO, and FTO/Cu_2_O/CuO/CuS photoelectrodes [0.5 Hz chopped illumination at 100 mW cm^−2^ AM 1.5 G]. (**a**) Complete experimental window. (**b**) Details of the curve after 1000 s.

To observe the differences in photostability, the surface potential was maintained constant at 0 V versus RHE while intermittently illuminating the sample. Figure 8 showed the difference in photocurrent stability over time for the Cu_2_O, Cu_2_O/CuO, and Cu_2_O/CuO/CuS samples. Cuprous oxide photoelectrode exhibited a quick quenching of the photocurrent density, in agreement with the previous considerations made on its stability. During the test, the semiconductor was indeed reduced to the metallic state, progressively diminishing the amount of photoactive material available, resulting in a photocurrent density of − 0.025 mA cm^−2^ after 1800 s of chopped illumination (Fig. 8b). The double and triple heterostructures showed higher photostability than bare cuprous oxide as expected from the LSV investigation. In particular, the sulfurized sample showed photocurrent of − 0.51 mA cm^−2^ at 500 s and − 0.31 mA cm^−2^ at 1800 s, corresponding respectively to 82% and 15% higher than the oxidized sample. The majority of the photocurrent was lost after about 10 min and subsequently stabilized at values higher than those of Cu_2_O/CuO heterostructure. After the polarization test, the sample surface showed a similar morphology to the as-synthesized one (Fig. S8) while the surface composition was found to be richer in copper, as shown by EDS (Fig. S9), suggesting that a part of the photocurrent was associated to self-reduction rather than hydrogen evolution reaction. Although the performance improvement was visible, photodegradation occurred and the results were in contrast with a previous report on Cu_2_O/CuO/CuS heterostructure^21^. In this regard, the employment of a substrate (Cu) could result in a more favorable energy level alignment due to the higher work function value than FTO.

**Figure 9:**
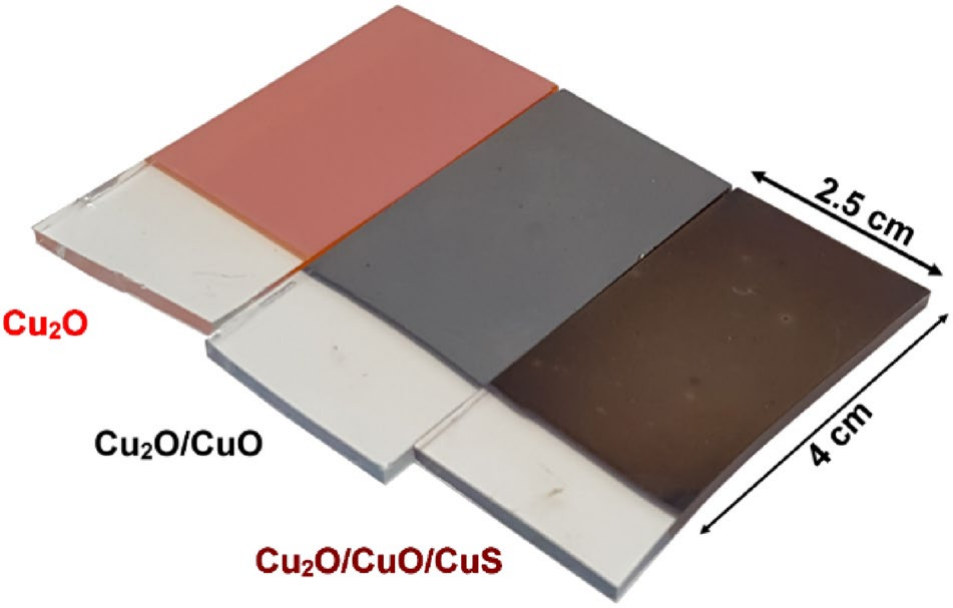
Picture of 10 cm^2^ samples.

**Figure 10:**
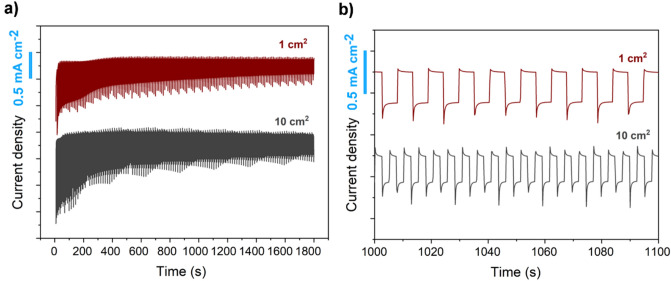
Photoelectrochemical test at fixed potential (0 V vs RHE) under chopper illumination for 1 cm^2^ and 10 cm^2^ FTO/Cu_2_O/CuO/CuS photoelectrodes [0.5 Hz chopped illumination at 100 mW cm^−2^ AM 1.5 G]. (**a**) Complete experimental window. (**b**) Details of the curve after 1000 s.

To demonstrate the feasibility of the approach proposed, a large area photoelectrode of about 10 cm^2^ was fabricated. The samples showed to be macroscopically homogeneous at each synthesis step showing a clear color change, distinguishable at naked-eye (Fig. 9). More importantly, to evaluate the scalability of the fabrication proposed, the photoelectrochemical performances of the large area photoelectrode were compared to a smaller area device of 1 cm^2^. The amperometric tests (Fig. 10) showed no differences between the two samples, indicating that the modification of the cupric oxide layer through reactive annealing was reliable as demonstrated to homogeneously modify the overall surface oxide surface.

## Conclusions

Sulfurization treatment has been proved to be an effective way to modify the surface of Cu_2_O/CuO heterostructure. The reactive annealing resulted in the formation of a three-layered heterostructure having a CuS overlayer, homogeneously covering the CuO surface. Thermal oxidation and reactive annealing showed the formation of CuO and CuS layers respectively, having no preferential orientation contrary to the (111) preferentially oriented Cu_2_O underneath layer. A progressive reduction of surface roughness occurred after the treatments and the characteristic pyramidal texture of Cu_2_O was flattened. Photoelectrochemical data showed that such modification improved the stability, reducing photodegradation, and increased the maximum photocurrent delivered (− 1.38 mA cm^−2^ at 0 V vs RHE under 100 mW cm^−2^ AM 1.5 G illumination). The fabrication of a 10 cm^2^ sample demonstrated that the proposed method is scalable and promising for potentially large-area and low-cost devices.

## Supplementary information

Supplementary Figures.
