# Supplementary material for: Modification of large area Cu2O/CuO photocathode with CuS non-noble catalyst for improved photocurrent and stability
Source: Sci Rep. 2020 Oct 30;10:18730. doi: 10.1038/s41598-020-75700-7 (PMC7603340; doi:10.1038/s41598-020-75700-7)
Supplement: Supplementary file 1 — Supplementary Figures. [file 41598_2020_75700_MOESM1_ESM.docx]

Supplementary Information

**Modification of large area Cu_2_O/CuO photocathode with CuS non-noble catalyst for improved photocurrent and stability**

G. Panzeri^a^, M. Cristina^a^, M. S. Jagadeesh^b^, G. Bussetti^b^, L. Magagnin^a^

^a^ Dipartimento di Chimica, Materiali e Ingegneria Chimica Giulio Natta

Politecnico di Milano, 20131 Milano, Italy

^b^ Dipartimento di Fisica, Politecnico di Milano, 20133 Milano, Italy

* Corresponding author: luca.magagnin@polimi.it

| **a**  **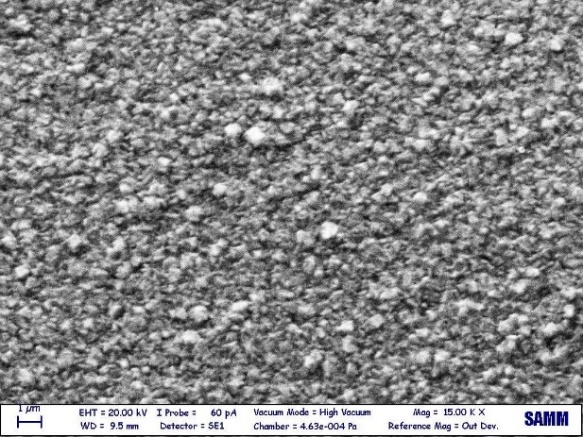** | b  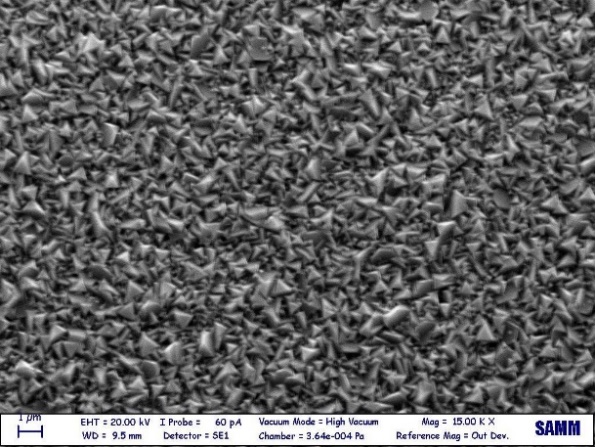 |
| --- | --- |
| c  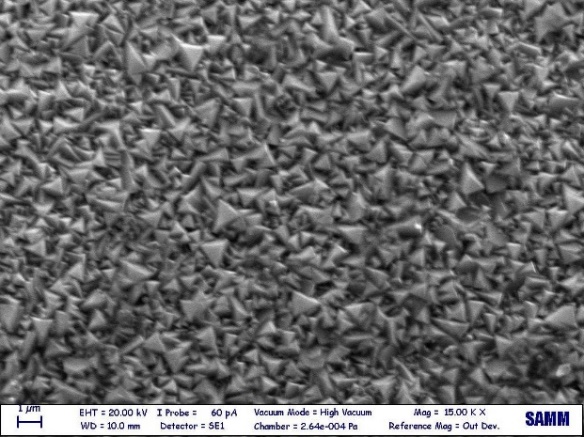 | d  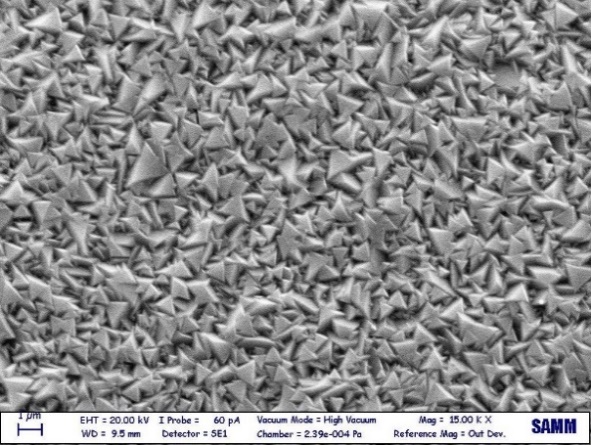 |

Fig. S1 SEM of Cu_2_O film. (a) 0.5 C/cm^2^. (b) 0.75 C/cm^2^. (c) 1 C/cm^2^. (d) 1.25 C/cm^2^.







Fig. S2 CuO (tenorite) ICSD-16025

Fig. S3 Normalized XRD spectra of Cu_2_O/CuO samples (0.75 C/cm^2^ Cu_2_O) at increasing annealing time: 0.5, 1, 1.5 and 2 h.


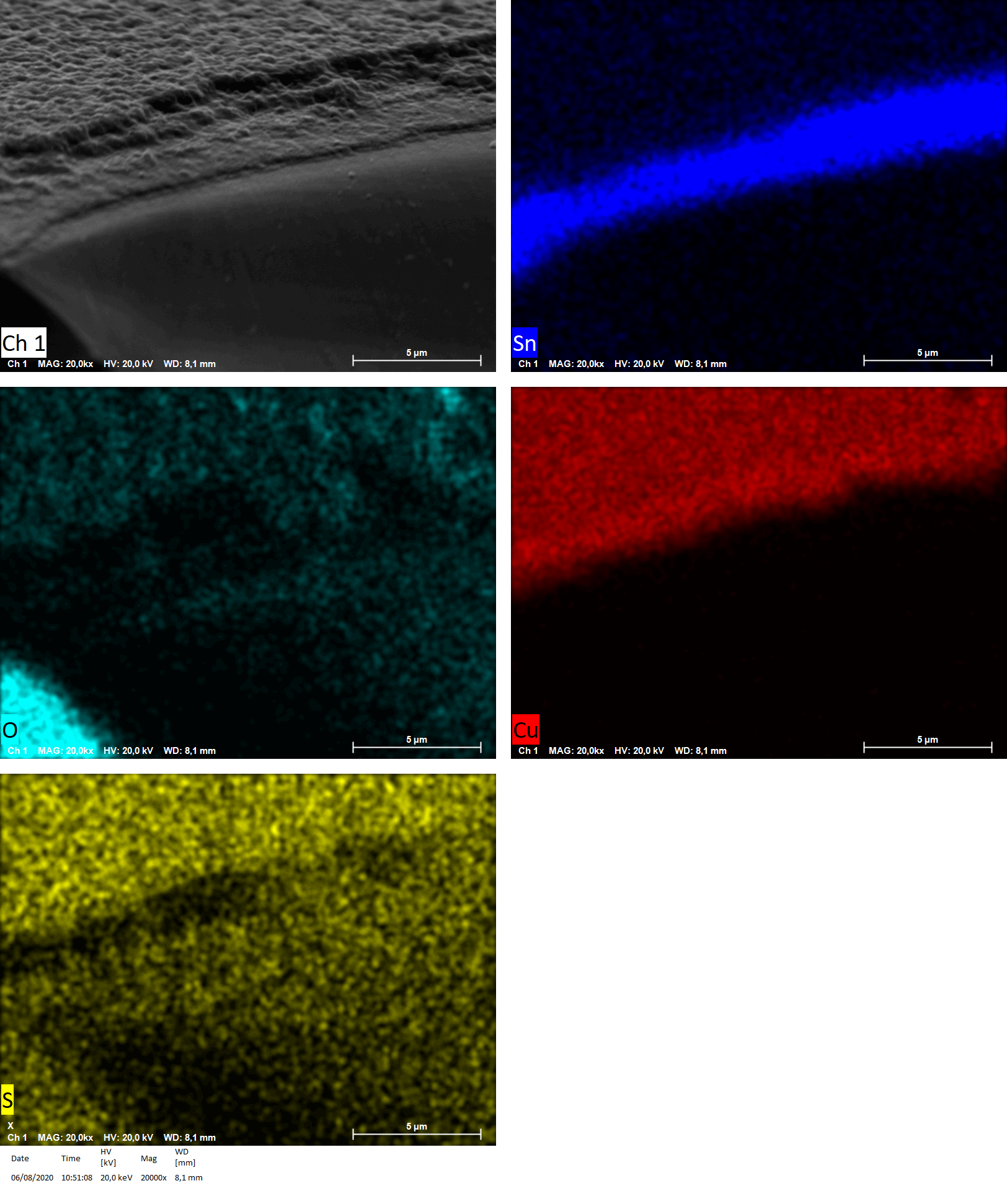


Fig. S4 SEM and EDS elemental mapping of FTO/Cu_2_O/CuO/CuS fracture surface


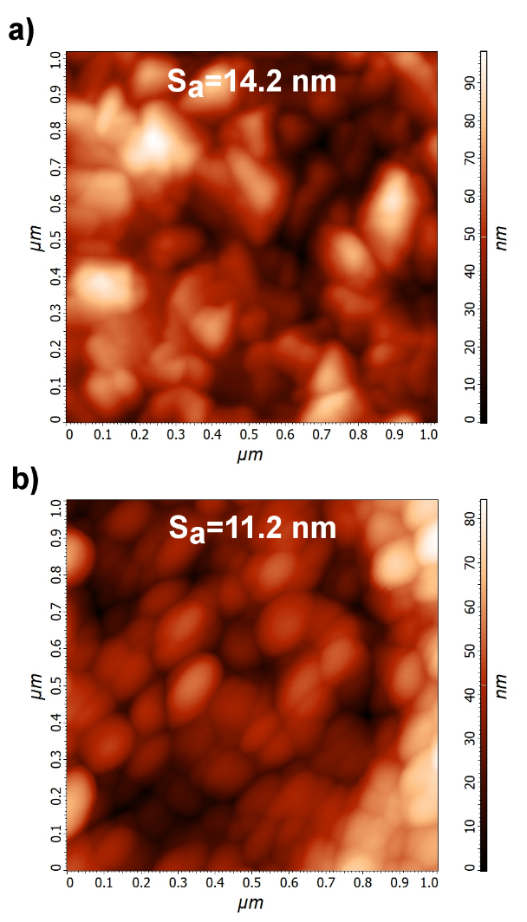


Fig. S5 AFM of (a) FTO/Cu2O/CuO and (b) FTO/Cu2O/CuO/CuS photoelectrodes





Fig. S6 LSV of Cu_2_O photoelectrodes in 0.5 M Na_2_SO_4_ at pH 5.

**

**

Fig. S7 LSV of Cu_2_O/CuO photoelectrodes in 0.5 M Na_2_SO_4_ at pH 5.


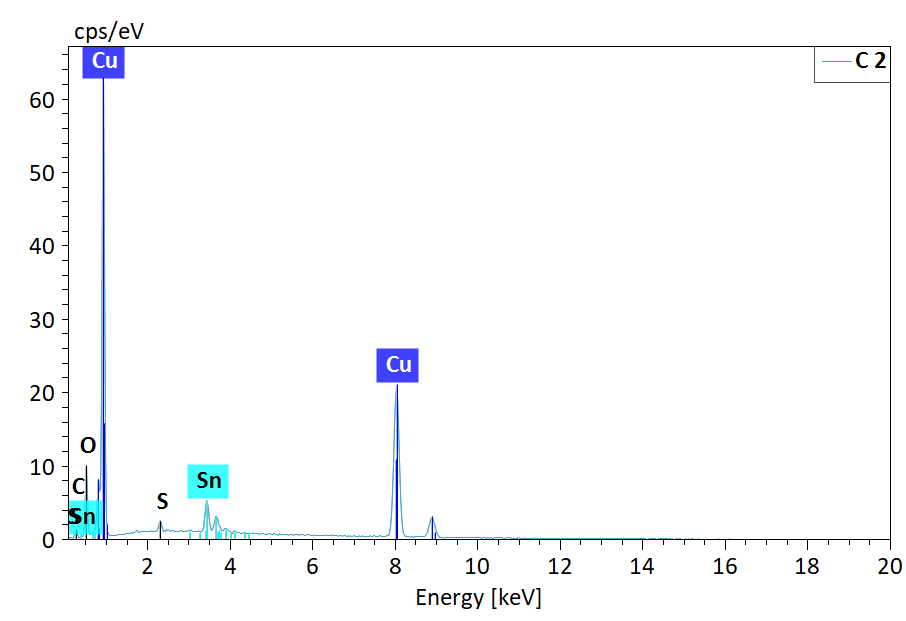
**
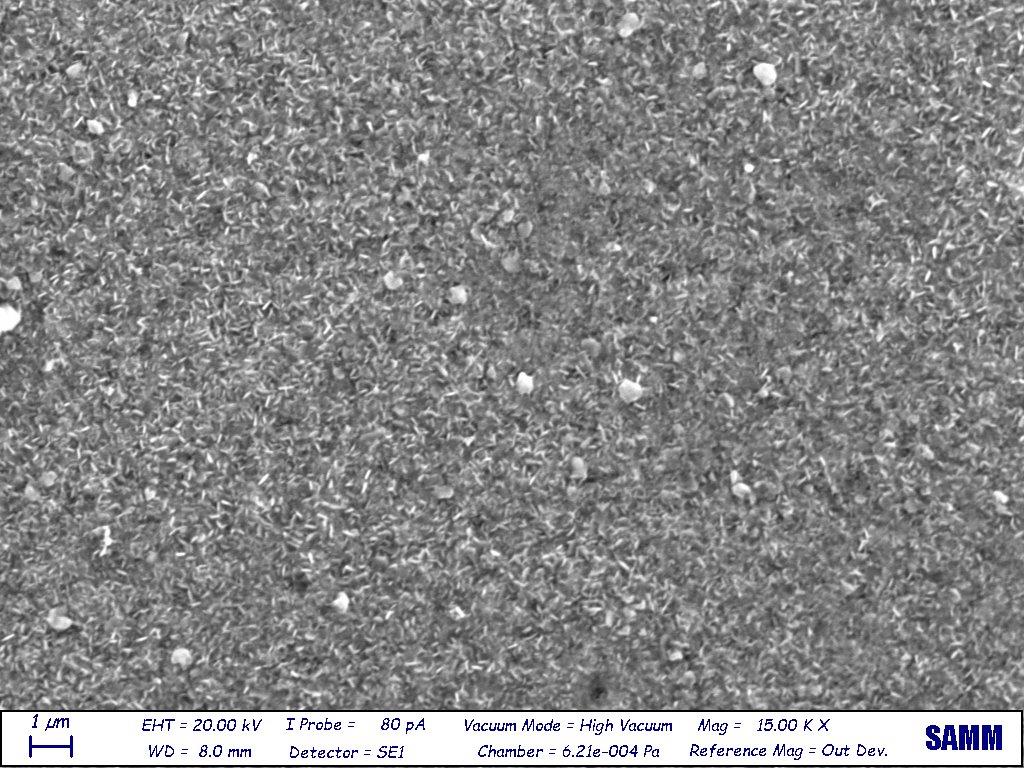
**

Fig. S9 EDS spectrum of FTO/Cu_2_O/CuO/CuS film after the potentiostatic (0 V vs RHE) photoelectrochemical test [0.5 Hz chopped illumination at 100 mW/cm^2^ AM 1.5 G].

Fig. S8 SEM of FTO/Cu_2_O/CuO/CuS film after the potentiostatic (0 V vs RHE) photoelectrochemical test [0.5 Hz chopped illumination at 100 mW/cm^2^ AM 1.5 G].
